# Delineation of Novel Autosomal Recessive Mutation in *GJA3* and Autosomal Dominant Mutations in *GJA8* in Pakistani Congenital Cataract Families

**DOI:** 10.3390/genes9020112

**Published:** 2018-02-20

**Authors:** Shazia Micheal, Ilse Therésia Gabriëla Niewold, Sorath Noorani Siddiqui, Saemah Nuzhat Zafar, Muhammad Imran Khan, Arthur A. B. Bergen

**Affiliations:** 1 Department of Clinical Genetics, Academic Medical Centre, Meibergdreef 9, 1105 AZ Amsterdam, The Netherlands; s.micheal@amc.uva.nl (S.M.); i.t.niewold@amc.uva.nl (I.T.G.N.); 2 Department of Pediatric Ophthalmology and Strabismus, Al-Shifa Eye Trust Hospital Jhelum Road, Rawalpindi 46000, Pakistan; sorathnoorani@yahoo.com (S.N.S.); saemahsaqib@yahoo.co.uk (S.N.Z.); 3 Department of Human Genetics, Radboud University Medical Centre, 6525 GA Nijmegen, The Netherlands; MuhammadImran.Khan@radboudumc.nl; 4 Department of Ophthalmology, Academic Medical Centre, 1105 AZ Amsterdam, The Netherlands; 5 Netherlands Institute for Neuroscience, (NIN-KNAW), 1105 AZ Amsterdam, The Netherlands

**Keywords:** congenital cataract, homozygosity mapping, sanger sequencing, proband, *GJA3* gene, *GJA8*, mutation, segregation

## Abstract

Congenital cataract is a clinically and genetically heterogeneous disease. The present study was undertaken to find the genetic cause of congenital cataract families. DNA samples of a large consanguineous Pakistani family were genotyped with a high resolution single nucleotide polymorphism Illumina microarray. Homozygosity mapping identified a homozygous region of 4.4 Mb encompassing the gene *GJA3*. Sanger sequence analysis of the *GJA3* gene revealed a novel homozygous variant c.950dup p.(His318ProfsX8) segregating in an autosomal recessive (AR) manner. The previously known mode of inheritance for *GJA3* gene mutations in cataract was autosomal dominant (AD) only. The screening of additional probands (*n* = 41) of cataract families revealed a previously known mutation c.56C>T p.(Thr19Met) in *GJA3* gene. In addition, sequencing of the exon-intron boundaries of the *GJA8* gene in 41 cataract probands revealed two additional mutations: a novel c.53C>T p.(Ser18Phe) and a known c.175C>G p.(Pro59Ala) mutation, both co-segregating with the disease phenotype in an AD manner. All these mutations are predicted to be pathogenic by in silico analysis and were absent in the control databases. In conclusion, results of the current study enhance our understanding of the genetic basis of cataract, and identified the involvement of the *GJA3* in the disease etiology in both AR and AD manners.

## 1. Introduction

Congenital cataract is the leading cause of visual impairment and blindness worldwide during infancy and early childhood. The disease is characterized by opacities of the lens and decreased visual acuity. The prevalence of congenital cataract was estimated to be 1–6 cases per 10,000 live births in developed countries, and 5–15 cases per 10,000 in the underdeveloped countries [1]. Worldwide estimates show that approximately 200,000 children every year are affected by lifelong vision impairment due to cataract [2]. 

Multiple factors are known to be involved in the pathogenesis of the disease. Among them, genetic factors are the most common, and explain approximately 50% of the cases. The disease segregates in a typical Mendelian manner, i.e., autosomal dominant (AD), autosomal recessive (AR) and X-linked. However, autosomal dominant transmission with high penetrance seems to be the most frequent [3]. In congenital cataract patients, early diagnosis is very important to treat the disease by surgically removing the visually significant cataracts and to achieve good vision. Nowadays, the most frequent surgical method used for the treatment and management of childhood cataracts is micro-incision cataract aspiration together with primary intraocular lens (IOL) implantation [4].

Currently, over 48 genes have been identified underlying congenital cataract. About 50% of the families have pathogenic mutations in crystalline genes; almost 25% have changes in the connexin genes [5]; and the remainder have causative mutations in genes such as aquaporin (*MIP*) [6], beaded filament structural protein 2 (*BFSP2*) [7], paired-like homeodomain 3 (*PITX3*) [8], avian musculoaponeurotic fibrosarcoma (*MAF*) [9], heat shock transcription factor 4 gene (*HSF4*) [10], lens intrinsic membrane protein (*LIM2)* [11], glucosaminyl (*N*-acetyl) transferase 2 (*GCNT2)* [12] as well as many others, as delineated in the Cat-Map database [13].

In this study, we report for the first time a homozygous novel mutation c.950dup p.(His318ProfsX8) in the homozygous region encompassing gap junction alpha 3 (*GJA3)* gene in a Pakistani family with a recessively inherited congenital cataract. In addition, sequencing of *GJA3* and *GJA8* genes in 41 congenital cataract probands revealed one known mutation in *GJA3* gene inherited dominantly and two mutations in *GJA8* gene (one novel and one known) segregating in an autosomal dominant fashion in cataract families.

## 2. Materials and Methods 

The participants were recruited at the pediatric ophthalmology department of Al-Shifa Eye Trust Hospital, Rawalpindi, Pakistan. The study was approved by the Institutional Review Board of the Al-Shifa Eye Trust Hospital (Rawalpindi, Pakistan), and adhered to the tenets of the Declaration of Helsinki, with the approval code PK2014:102. Written informed consent was obtained for study participation from the participants and/or their parents, as appropriate. Comprehensive, ocular, medical, and family histories were obtained from the parents/available family member. Detailed ophthalmic examination was performed for both affected and unaffected individuals of families. Blood samples were collected from affected and unaffected siblings, and from the parents. Genomic DNA was extracted using QIAGEN DNA Blood Midi Kit (QIAGEN, Germantown, Maryland, USA).

Genomic DNA samples of two affected and three unaffected family members of a large consanguineous cataract family were genotyped with Affymetrix 250K single nucleotide polymorphism (SNP) microarray (Affymetrix: Santa Clara, CA, USA), and genotype data were analyzed for homozygous regions using the online mapping tool HomozygosityMapper (http://www.homozygositymapper.org/). Haplotypes of the affected and unaffected members of the family were compared to find the identical homozygous regions among the affected individuals of the family. 

The coding exons and intronic boundaries of *GJA3* (NM_021954) gene were amplified in a family with the homozygous region encompassing the *GJA3* gene. Both *GJA3* and *GJA8* (NM_005267) genes were sequenced in probands of 41 additional cataract families. Primers were designed using Primer 3 (http://bioinfo.ut.ee/primer3-0.4.0/) (primer sequences and polymerase chain reaction (PCR) conditions are available on request). PCR products were analyzed on 2% agarose gels followed by Sanger sequencing using ABI BigDye chemistry (Applied Biosystems Inc., Foster City, CA, USA), and were processed through an automated ABI 3730 Sequencer (Applied Biosystems, Inc.). Sequences obtained were aligned with the reference sequence using CodonCode Aligner (version 6.1) (CodonCode Co., Centerville, MA, USA). Intra-familial segregation analysis was performed upon the identification of variants among the probands in the *GAJ3* and *GJA8* genes.

Pathogenicity of missense variants was evaluated by publically available tools including PhyloP, Grantham, polymorphism phenotyping v-2 (PolyPhen-2) (version 2.1.0 r367) (http://genetics.bwh.harvard.edu/pph2/), MutationTaster (http://www.mutationtaster.org/), and sorting intolerant from tolerant (SIFT, http://sift.bii.a-star.edu.sg/) to predict the functional impact of the sequence variants on the encoded protein. The amino acid sequences were obtained from protein sequence database UniProt (http://www.rcsb.org/pdb/protein/Q6P2Q9) from different species to check the amino acid conservation. Kalign (2.0) was used for the multiple nucleotide sequence alignment.

## 3. Results

### 3.1. Clinical Findings

**Figure 1:**
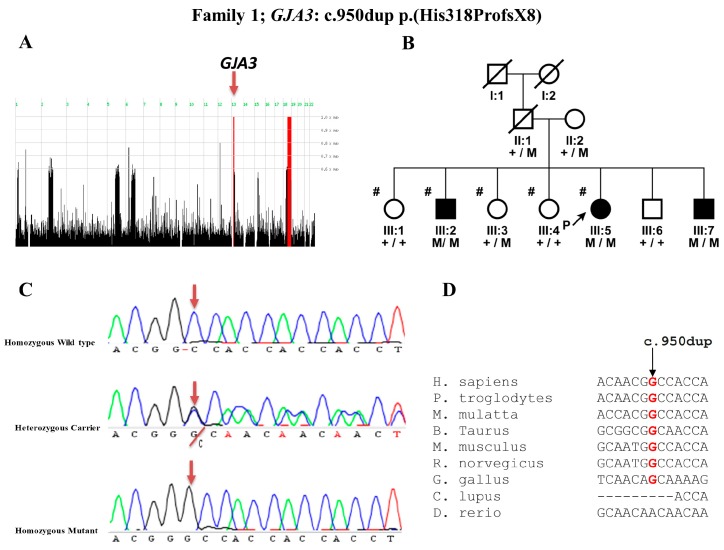
Homozygosity mapping and mutation analysis of Family 1: (**A**) homozygosity mapping results indicating homozygous region (red line) on chromosome 13 encompassing a Gap junction alpha 3 (*GJA3*) gene; (**B**) pedigree of a family with congenital cataract, and segregation of a novel mutation c.950dup p.(His318ProfsX8) in the *GJA3* gene; (**C**) DNA sequence chromatogram of *GJA3* for the normal (+/+), heterozygous carrier (+/M), and affected individuals (M/M); and (**D**) nucleotide conservation among the orthologues.

In Family 1, Proband (III:5) was a two-year-old boy presented with squinting eyes (Figure 1B). His corneal diameter was 11 mm horizontally and 10.5 mm vertically in both eyes measured with caliper under general anesthesia before surgery. Nuclear cataract was noted in both the eyes. All the affected individuals of the family after cataract surgery developed secondary glaucoma phenotype with intraocular pressure (IOP) > 22 mmHg and cup-to-disc ratio (CDR) > 0.7 which resulted in severe visual impairment. The heterozygous carrier individuals of the family which includes parents (II:1 and II:2) and unaffected siblings (III:1, III:3, III:4 and III:6) had normal crystalline lenses on clinical examination. 

In Family 2, proband (II:1) was a three-year-old boy with bilateral congenital nuclear cataracts with the central, dense nuclear lens opacity. The proband’s mother (I:2) also suffered from bilateral lens opacities. 

The pedigree of Family 3 comprised three generations with four affected and three unaffected individuals. The proband (III:4) was a three-month-old boy presented with leucocoria. He had bilateral central cataract, nystagmus and amblyopia. Intraocular pressure was 8 mm Hg in both eyes. Lensectomy was performed in both eyes and visual rehabilitation was done using aphakic spectacle correction of +20 diopter sphere (DS) in the right and +21DS in the left eye. His corrected vision on last follow-up was 6/30 in the right and 6/19 in the left eye. His mother, aunt and grandmother had a history of bilateral cataract. His elder sister also had congenital cataract shortly after birth, and had undergone cataract extraction at around three years of age.

The large Family 4 also consisted of three generations with four affected and one unaffected individual. The proband (III:1), his mother, aunt, and grandfather all presented with bilateral nuclear cataract. They all had bilateral surgery for cataract extraction. 

### 3.2. Mutation Detection in GJA3 Gene

In Family 1, we found two homozygous chromosomal regions among the two affected individuals of the family. One 4.4 Mb region was located on chromosome 13 containing the previously identified gene (*GJA3*) implicated in cataract (Figure 1A). Sanger sequencing of the coding exons of *GJA3* gene revealed a novel homozygous variant c.950dup p.(His318ProfsX8) co-segregating with the disease phenotype within the family in AR manner (Figure 1B, C). This duplication shifts the conceptual reading frame of the protein leading to a premature chain termination. Nucleotide sequence alignment shows that there was no gap between the nucleotides among different orthologous species (Figure 1D). This indicates that the duplication of the “G” nucleotide at the c.950dup position is not tolerated. 

**Figure 2:**
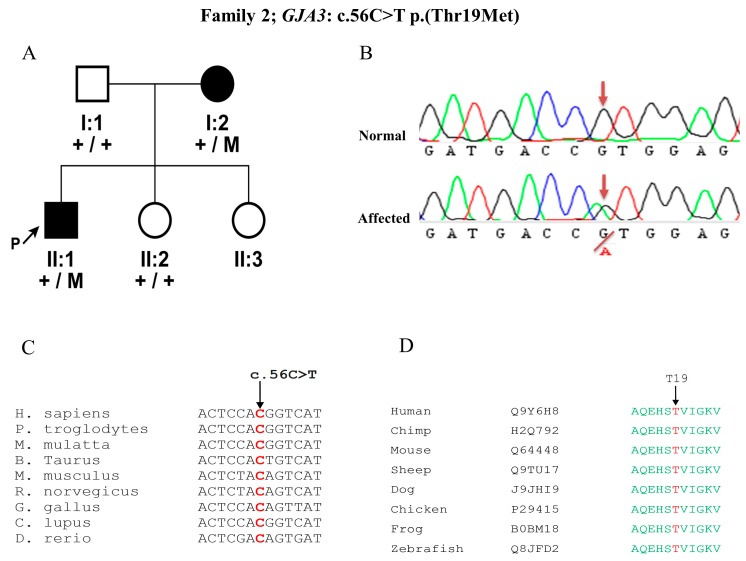
Pedigree of a Family 2 with dominant *GJA3* mutation: (**A**) Family with congenital cataract, showing segregation of a mutation c.56C>T p.(Thr19Met) in the *GJA3* gene. Normal individuals are represented with +/+ and affected individuals with the +/M symbol for the mutation. (**B**) Sanger sequencing DNA chromatogram of *GJA3* for the normal and affected individuals. (**C**, **D**) Nucleotide and amino acid conservation in orthologous species for the c.56C>T p.(Thr19Met). The wild type nucleotide (C) and amino acid (T) are represented with arrow and in red color.

In Family 2, Sanger sequencing of the proband revealed a previously described mutation in the *GJA3* gene c.56C>T p.(Thr19Met), segregating in our family with the disease phenotype in an AD manner (Figure 2A, B). This particular variant was located in the connexin domain of the protein and was predicted to be disease causing, deleterious, damaging by MutationTaster, SIFT, and Polyphen-2, respectively, with a PhyloP score of 6.18 (indicates nucleotide conservation) (Figure 2C) and Grantham score of 81, as threonine is a highly conserved amino acid among different species (Figure 2D).

### 3.3. Mutation Detection in GJA8 Gene

**Figure 3:**
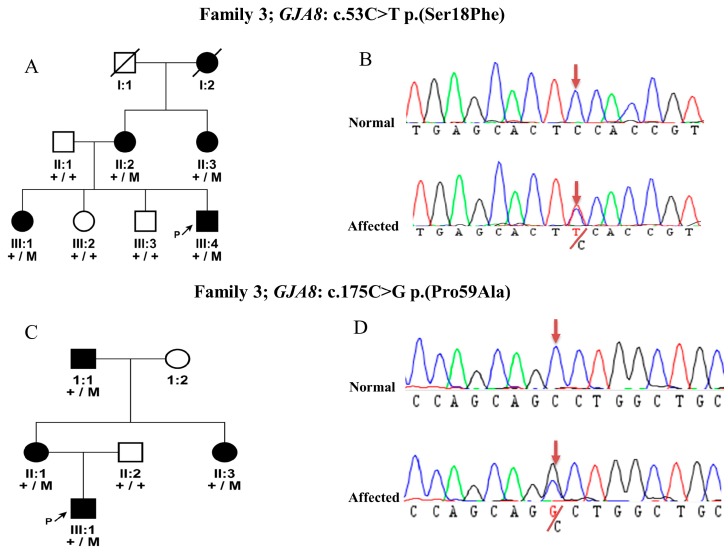
Pedigrees of two families with *GJA8* mutations: (**A**) pedigree of Family 3 with congenital cataract, and segregation of a novel mutation c.53C>T p.(Ser18Phe) in the *GJA8* gene; (**B**) DNA sequence chromatogram of c.53C>T p.(Ser18Phe) mutation; (**C**) Family 4 segregation of a mutation c.175C>G p.(Pro59Ala) in the *GJA8* gene; and (**D**) DNA sequence chromatogram of c.175C>G p.(Pro59Ala) mutation in *GJA8.*

In Family 3, direct sequencing of the probands of cataract families identified a novel missense variant c.53C>T p.(Ser18Phe) in the *GJA8* gene. This variant segregate with the disease phenotype in the family in AD manner (Figure 3A, B). The Ser18Phe variant is located in the connexin domain of the protein. The amino acid residue Ser18 is highly conserved among different species. It is predicted to be disease causing, deleterious, and damaging by MutationTaster, SIFT, and Polyphen-2, respectively, with a Grantham score of 155 and a PhyloP score of 5.86, which indicates both amino acid and nucleotide conservation among different species.

In Family 4, a missense variant c.175C>G p.(Pro59Ala) was identified in *GJA8,* and was found to be segregating with the disease phenotype dominantly (Figure 3C, D). This variant was previously known for its association with cataract and is localized in the connexin domains. This change in amino acid was also predicted to be disease causing by in-silico predictions with a Grantham score of 27 and a PhyloP score of 5.94.

**Figure 4:**
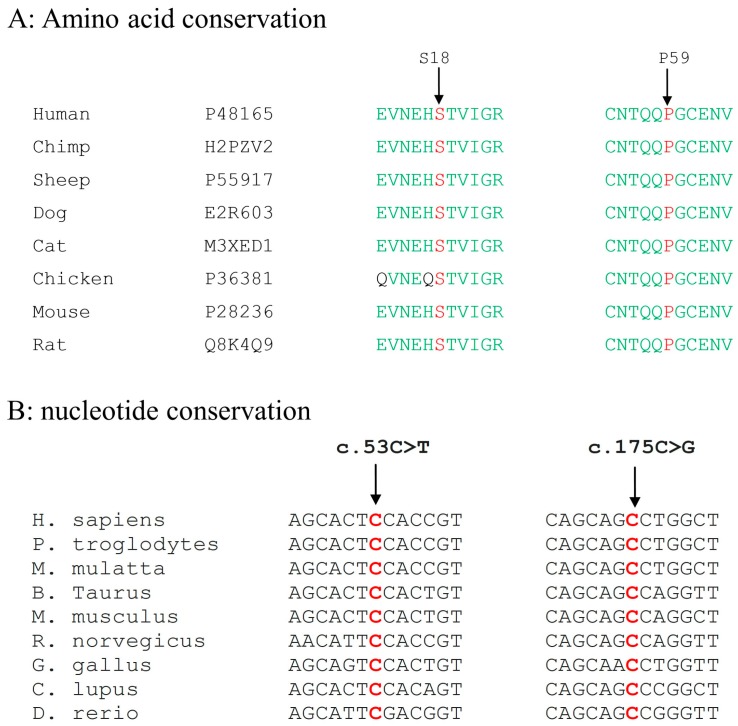
Multiple sequence alignment of *GJA8* orthologues: (**A**) mutated amino acids p.(Ser18Phe) (S), and p.(Pro59Ala) (P) are indicated with an arrow and in red color; and (**B**) nucleotide conservation among the orthologous species is indicated with arrow and the wild type nucleotide is represented in red color.

Both the wild type nucleotides at positions c.53C, and c.175C as well as amino acids at positions Ser18 and Pro59 are highly conserved among the orthologous species (Figure 4). 

All variations identified in the *GJA3* and *GJA8* genes have been excluded from the population matched 100 controls by performing Sanger Sequencing of the particular exons. All these variants are absent in the publically available Gnomad database for *GJA3* (http://gnomad.broadinstitute.org/gene/ENSG00000121743) and *GJA8* (http://gnomad.broadinstitute.org/gene/ENSG00000121634) genes.

## 4. Discussion

Previously, multiple genes have been implicated in cataract [13]. In this study, we have focused on *GJA3* and *GJA8* underlying human cataract frequent mutations in these genes explaining about 25% of the patients [5]. We sequenced both genes in our current panel of probands of Pakistani families. In this study, we report a novel homozygous mutation c.950dup p.(His318ProfsX8) in the *GJA3* gene causing autosomal recessive cataract in a large Pakistani family. Previously, mutations in *GJA3* in animal models were associated with recessively inherited form of cataract [14], but, in humans, only autosomal dominant *GJA3* mutations have, so far, been implicated in the disease [15,16]. 

In case of our Family 1, the proband was initially recruited with a (secondary) glaucoma phenotype. Taking into account the recessive inheritance pattern of family, homozygosity analysis was performed. However, later, clinical re-examination of all the affected members of the family revealed autosomal recessive cataract as a primary phenotype. Since the homozygous region identified also encompassed the *GJA3* gene, we subsequently focused on Sanger sequencing of this cataract candidate gene to discover the novel homozygous mutations, segregating in the family with the disease. Previously, pathogenic mutations have been found all over the *GJA3* protein [17]. Notably, our p.(His318ProfsX8) homozygous variant is present in the middle of the carboxy-terminal domain (CT) of the *GJA3* where previously no other dominant change has been identified. This CT domain is specific for the connexin isotypes and is important for the post-translational modifications and for interactions with other protein partners [18]. This duplication creates a frame shift starting at codon His318. The new reading frame ends in a stop codon 7 positions downstream. Thus, the protein will be smaller in length with mistranslation of few amino acids. Due to the presence of early stop, the abnormal mRNA might be subjected to nonsense-mediated decay. The recessive mutations are mostly responsible for causing severe disease phenotypes. In the current study, individual III:2 of Family 1, with the recessive mutation in the *GJA3* gene indeed suffered from severe secondary glaucoma after cataract surgery with high cup-to-disc ratio of 0.8 in both eyes, and intraocular pressure was 28 mmHg and 34 mmHg for the right and left eye respectively. Upon the follow-up of all the affected individuals of the family, secondary glaucoma was observed with IOP > 22 mmHg and CDR > 0.7. The severe secondary glaucoma phenotype in all the affected individuals could be one of the outcomes of the recessive mutation in *GJA3* in this particular family. Similarly, in the Indian family, a recessive mutation c.670insA p.(Thr203AsnfsX47) was reported in the *GJA8* gene with an additional secondary phenotype of nystagmus and amblyopia due to severe visual impairment [19]. However, the majority of mutations reported in the *GJA8* were inherited in an autosomal dominant manner. In the current family with *GJA3* mutation and previous families with *GJA8* mutations recessive inheritance was associated with the severe phenotype together with the additional secondary phenotypes.

Approximately 38–40 *GJA3* mutations were previously implicated in dominantly inherited cataract. In one of our families, a previously known mutation c.56C>T p.(Thr19Met) was identified, which is also inherited in an autosomal dominant manner. In the current study, the proband (II:1) having this mutation (Family 2) was presented with nuclear cataract at the age of three years. Interestingly, this particular mutation has also been reported in an Indian family with posterior polar cataract. The proband of the Indian family was presented also at the age of three years [20]. In addition, in a rat model suffering from autosomal recessive congenital nuclear cataract, a missense mutation in *gja3,* p.Glu42Lys, has been reported [14]. A homozygous deletion of the *gja3* in a knockout mouse resulted in congenital cataract [21]. These findings suggest that at least one cataract mutation may result in a pleiotropic phenotype, which contributes to the understanding of the complexity of cataract genetics. 

In addition to *GJA3* variants, we also identified pathogenic changes in *GJA8* gene which include a previously known c.175C>G p.(Pro59Ala) and a novel c.53C>T p.(Ser18Phe) mutation. The p.(pro59Ala) mutation was previously reported in a Chinese family presented with the congenital nuclear cataract [22]. Interestingly, the phenotype and age of onset of the cataract in the Pakistani family was similar to a Chinese family. In a previous study, the functional effect of the p.(Pro59Ala) change was characterized by stably transfecting recombinant DNA constructs into Hek293 cells. In contrast to the wild type protein, the *GJA8* protein with the pathogenic variant failed to form gap junction plaques at appositional membrane. The mutation had also an effect on the cell viability, growth and proliferation [22]. 

The novel p.(Ser18Phe) mutation is also predicted to be a pathogenic change and is located close to previously known mutations p.(Leu7Pro) and p.(Arg23Thr) at the N-terminal domain of *GJA8* [23,24]. In addition, the p.(Arg23Thr) change was previously functionally characterized in vitro. HeLa cells were stably transfected with the mutant and wild types. Decreased intercellular communication was reported in the presence of mutated amino acid [24]. In p.(Arg23Thr) a basic arginine has been replaced with the polar uncharged threonine amino acid. Since the p.(Ser18Phe) novel mutation identified in a Pakistani family is located in proximity to the previous mutation and polar uncharged serine has been replaced with the hydrophobic phenylalanine amino acid, it could be that, due to the p.(Ser18Phe) mutation, intercellular communication is affected. This mutation may affect the secondary/tertiary structure of the protein or the interaction of the *GJA8* with the other proteins.

In previous studies in mice, it has been observed that the complete knockout of either of the two connexins; *GJA3* or *GJA8,* resulted in cataract formation and decreased lens growth. These mutated connexins affect the formation of primary and secondary lens fiber cells which resulted in the cataract phenotype [25]. The lens is an avascular organ and due to the lack of vasculature it is dependent on the formation of a network of gap junctions. Lens tissues express three types of connexins: CX43 (GJA1), CX46 (*GJA3*) and CX50 (*GJA8*). *GJA3* (CX46) and *GJA8* (CX50) are connexins involved in the formation of these networks. Gap junctions are important in the intracellular communication essential for the cell survival, function and maintenance of the lens homoeostasis and transparency [25]. Both connexins are part of a large multigene family comprising of 21 members. All connexins, consists of four transmembrane domains (M1–M4), two extracellular loops, a cytoplasmic loop, NH_2_-terminal, and COOH-terminal domains. They all form connexin hemi-channels important for the permeation of ions, as well as small metabolites such as adenosine triphosphate (ATP), cyclic adenosine monophosphate (cAMP), inositol triphosphate (IP3) and glutamate. 

The expression pattern of CX43, CX46 and CX50 connexins in the lens is highly dynamic. *GJA3* (CX46) is significantly upregulated during the differentiation of the lens epithelial-to-fiber cell. *GJA3* plays a vital role in the regulation of several cellular processes such as cell growth, proliferation, differentiation, and apoptosis [26,27]. *GJA8* (CX50) is also highly expressed in lens fiber cells and epithelial cells. It is involved in the growth and differentiation of the lens tissues. The higher expression was observed during the lens epithelial fiber cell differentiation [28]. In addition to gap junction formation, these connexins are also involved in the regulation of the cell cycle, especially *GJA8* (CX50) which is involved in the cell cycle arrest [29]. 

Apparently, the lens has a controlled mechanism for the opening and closing of the connexin hemi-channels, but, in the case of mutated connexins, it may be they are inefficient to prevent the openings of the hemi-channels and to decrease the intake by them. Thus, the presence of c.56C>T p.(Thr19Met), c.427G>A p.(Gly143Arg) in *GJA3* [25,26] and c.137G>T p.(Gly46Val) in *GJA8* [30] and the novel mutations identified in current study may result in reduced gap junction channel formation and aberrant/increased hemi-channel activity and, eventually, cell death [31,32]. The in-silico analysis of rare variants identified in the current study (*GJA3* and *GJA8* genes) and reported in the discussion section predicted all these variants as deleterious and disease causing (Table 1). *GJA3* (CX46), *GJA8* (CX50) and CX43 (GJA1) have been reported vital for interconnecting the lens fiber and epithelial cells in humans [33]. 

Interestingly, mutations in yet another connexin member, *GJA1,* are associated both dominantly and recessively with oculodentodigital dysplasia (ODDD) including the ocular phenotypes of microphtalmia, microcornea, cataract and glaucoma [34]. In few patients with ODDD, iris anomalies and secondary glaucoma have also been reported [34,35]. Previous studies have reported both dominant and recessive modes of inheritance of cataract associated with both the *GJA1* in ODDD and *GJA8* in congenital cataract. This study is the first one reporting the recessive mode of inheritance for the *GJA3* gene mutations in cataract.

In summary, the results of the current study demonstrate a new inheritance pattern of *GJA3* gene mutations with cataract. We report a novel homozygous duplication p.(His318ProfsX8) in *GJA3* associated with nuclear cataract in a Pakistani family. In addition, we identified one novel mutation p.(Ser18Phe) in the *GJA8* gene inherited dominantly in a Pakistani family. The results of the current study help broaden the spectrum of mutations identified in *GJA3* and *GJA8* genes, and are of utmost importance for genetic counselling and family planning of parents at risk. Additional cataract families with the recessive inheritance should be screened for the mutations in *GJA3* gene. 

## Figures and Tables

**Table 1:** In-silico analysis of rare variants in *GJA3* and *GJA8* genes.

Gene ID	cDNA Position	Amino Acid Position	Study	phyloP	Grantham Score	SIFT	Mutation Taster	Poly Phen-2
*GJA3*	c.950dup	p.(His318ProfsX8)	Current	N/A	N/A	D	Disease causing	Damaging
	c.56C>T	p.(Thr19Met)	Current	6.18	81	D	Disease causing	damaging
	c.427G>A	p.(Gly143Arg)	Previous	5.86	125	D	Disease causing	damaging
	c.137G>T	p.(Gly46Val)	Previous	5.94	109	D	Disease causing	damaging
*GJA8*	c.53C>T	p.(Ser18Phe)	Current	5.86	155	T	Disease causing	damaging
	c.175C>G	p.(Pro59Ala)	Current	5.96	27	D	Disease causing	damaging
	c.20T>C	p.(Leu7Pro)	Previous	3.35	98	T	Disease causing	damaging
	c.68G>C	p.(Arg23Thr)	Previous	4.16	71	T	Disease causing	damaging

Foot Note: D; deleterious, T; Tolerated.
